# Overexpression of CERKL Protects Retinal Pigment Epithelium Mitochondria from Oxidative Stress Effects

**DOI:** 10.3390/antiox10122018

**Published:** 2021-12-19

**Authors:** Rocío García-Arroyo, Aleix Gavaldà-Navarro, Francesc Villarroya, Gemma Marfany, Serena Mirra

**Affiliations:** 1 Department of Genetics, Microbiology and Statistics, Avda. Diagonal 643, Universitat de Barcelona, 08028 Barcelona, Spain; rociogarciaarroyo@ub.edu; 2 CIBERER, Instituto de Salud Carlos III, 28029 Madrid, Spain; 3 Institut de Biomedicina-Institut de Recerca Sant Joan de Déu (IBUB-IRSJD), Universitat de Barcelona, 08028 Barcelona, Spain; aleixgavalda@ub.edu (A.G.-N.); fvillarroya@ub.edu (F.V.); 4 Department of Biochemistry and Molecular Biomedicine, Avda. Diagonal 643, Universitat de Barcelona, 08028 Barcelona, Spain; 5 CIBEROBN, Instituto de Salud Carlos III, 28029 Madrid, Spain; 6 DBGen Ocular Genomics, 08028 Barcelona, Spain

**Keywords:** retinitis pigmentosa, *CERKL*, retinal pigment epithelium, mitochondrial network, oxidative stress

## Abstract

The precise function of *CERKL*, a Retinitis Pigmentosa (RP) causative gene, is not yet fully understood. There is evidence that *CERKL* is involved in the regulation of autophagy, stress granules, and mitochondrial metabolism, and it is considered a gene that is resilient against oxidative stress in the retina. Mutations in most RP genes affect photoreceptors, but retinal pigment epithelium (RPE) cells may be also altered. Here, we aimed to analyze the effect of *CERKL* overexpression and depletion in vivo and in vitro, focusing on the state of the mitochondrial network under oxidative stress conditions. Our work indicates that the depletion of *CERKL* increases the vulnerability of RPE mitochondria, which show a shorter size and altered shape, particularly upon sodium arsenite treatment. *CERKL*-depleted cells have dysfunctional mitochondrial respiration particularly upon oxidative stress conditions. The overexpression of two human CERKL isoforms (558 aa and 419 aa), which display different protein domains, shows that a pool of CERKL localizes at mitochondria in RPE cells and that CERKL protects the mitochondrial network—both in size and shape—against oxidative stress. Our results support *CERKL* being a resilient gene that regulates the mitochondrial network in RPE as in retinal neurons and suggest that RPE cell alteration contributes to particular phenotypic traits in patients carrying *CERKL* mutations.

## 1. Introduction

Retinitis pigmentosa (RP) comprises a genetically heterogenous group of retinal degenerative diseases characterized by night blindness and progressive loss of vision due to photoreceptor degeneration. To date, more than 70 causative genes have been identified [1]. Mutations in *CERKL* (CERamide Kinase Like) have been reported to cause non-syndromic autosomal recessive RP [2] as well as cone-rod dystrophy (CRD) [3]. *CERKL* expression is highly complex, with more than 20 transcripts and several alternative promoters in human and mouse tissues [4,5].

This transcriptional complexity results in at least four CERKL isoforms displaying different protein domains [6]. CERKL isoforms have a dynamic subcellular localization and multiple functions: they act as a shuttle from the cytoplasm to the nucleus; are able to bind sphingolipids [7]; interact with antioxidant enzymes [8]; and regulate autophagy, and mitochondrial dynamics and metabolism [9,10]. Notably, CERKL has been also described as a RNA binding protein that localizes in polysomes, mRNA compact particles, stress granules (SG), and P-bodies under stress conditions [11]. Altogether, these findings point to *CERKL* being a resilient gene acting in multiple pathways to protect retinal photoreceptors against oxidative stress. Thus, mutations in CERKL most probably increase the sensitivity of retinal tissue to oxidative damage, resulting in cell death and retinal neurodegeneration [12].

Knowledge about *CERKL* function in the neuroretina has been growing during the last years with the obtention of several mouse models. Using CRISPR-Cas9 gene editing, our group generated a double heterozygous knock-down/knock-out, *Cerkl^KD/KO^*, mouse line, where some *Cerkl* expression was retained to ensure organism survival. Specifically, the *Cerkl^KD/KO^* retina expressed less than 20% of *Cerkl* compared with that of wild type [5]. In the retina, CERKL expression has been mostly described in rods, cones, and retinal ganglion cells [5]. Notably, most mutated genes that cause RP are also expressed in retinal pigment epithelium (RPE), and previous data from our lab showed a strong expression of CERKL in mouse RPE [5]. However, very little is known about the function of CERKL in RPE. RPE is a monolayer of post-mitotic cells located between the neuroretina and the choroid, playing an important role in retinal homeostasis. The functions of RPE include (a) helping renew outer segments by phagocytosis and degradation of spent discs of photoreceptor outer segments, (b) ensuring photoreceptor survival by supplying small molecules (amino acids, ascorbic acid, and D-glucose), (c) supporting the recycling of molecules associated with the visual cycle, (d) creating a firm barrier against choroidal blood-borne substances, and (e) protecting the outer retina from excessive high-energy light and light-generated reactive oxygen species (ROS). Consequently, RPE is shown to have an immense metabolic activity. Recent evidence suggests that mitochondrial damage and oxidative stress in the RPE may play important roles in RP pathogenesis [13].

Mitochondria are crucial organelles that provide energy to the cell because of oxidative phosphorylation. In addition, they are important to buffer calcium, to control the cell cycle, and to regulate apoptosis. It is estimated that 1–5% of ROS is generated by mitochondrial activity in physiological conditions [14]. An altered mitochondrial function caused by environmental or physiological changes generates loss of mitochondrial membrane potential, decreases oxidative phosphorylation, causes damage in mitochondrial DNA, and creates a vicious circle of ROS-generated ROS [15]. These perturbations induce reorganization of the mitochondrial network through changes in mitochondrial dynamics that consist of fission, fusion, transport, communication between organelles, and quality control mechanisms [16]. All these alterations can finally lead to programmed cell death. Importantly, RPE experiences a high-oxygen environment, which is exacerbated by the loss of rods in RP, thus creating a hyperoxic environment that is presumably hostile for the remaining cells [17]. One protective strategy in RP and other inherited retinal dystrophies is to augment the oxidative damage defense systems in both retina and RPE [15].

Here, we describe the CERKL-mediated cellular response to oxidative stress in RPE, focusing on mitochondria. By comparing the RPE of wild-type (WT) and *Cerkl^KD/KO^* (heretofore *KD*/*KO*) animals combined with in vitro assays, we study the impact of CERKL expression levels on RPE mitochondrial network organization and dynamics at basal conditions and under oxidative stress.

## 2. Materials and Methods

### 2.1. Animal Handling

Mouse tissue from WT and *Cerkl^KD/KO^* was obtained according to the ARVO statement for the use of animals in ophthalmic and vision research under the regulations of the Ethical Committee for Animal Experimentation (AEC) of the Generalitat of Catalonia according to the European Directive 2010/63/EU and other national laws. The procedures received institutional approval from the Universitat de Barcelona.

### 2.2. Transmission Electron Microscopy

Three-month-old C57BL6/J mice or twelve-month-old albino mice were transcardially perfused with cold fixative solution (2.5% glutaraldehyde and 2% PFA (paraformaldehyde) in 0.1 M phosphate buffer). Their eyes were removed, and the retinas were dissected and fragmented in 1 mm^3^ pieces. Retinal fragments were immersed in fixative solution (2.5% glutaraldehyde and 2% PFA in 0.1 M phosphate buffer) and incubated at 4 °C overnight. The retinal fragments were post-fixed in 1% osmium tetroxide 2% K_4_Fe(CN)_6_ in the dark for 2 h at 4 °C and rinsed in double-distilled water to remove the osmium. The retinal fragments were dehydrated in ascending concentrations of acetone, then infiltrated, and embedded in Epon (EMS). Blocks were obtained after polymerization at 60 °C for 48 h. Ultrathin sections of 60 nm in thickness were obtained using a UC6 ultramicrotome (Leica Microsystems, Vienna, Austria) and were stained with 2% uranyless and lead citrate. Sections were observed in a Tecnai Spirit 120 Kv TEM (FEI company, Eindhoven, The Netherlands), and images were acquired with a 1 k × 1 k CCD Megaview camera (Olympus Soft Imaging Solutions, Münster, Germany).

### 2.3. Whole-Mount RPE

For whole-mount RPE staining, RPE from adult mice were obtained as described in [18], placed on glass slides flattened by cutting the edges, fixed for 1 h in 4% paraformaldehyde, and rinsed with 1× PBS (3 × 5 min). The retinas were then incubated with Alexa Fluor 647 Phalloidin (Thermo Fisher Scientific, Rockford, IL, USA; A22287) for 1 h. Retinas were washed with 1X PBS (3 × 5 min) and mounted with Fluoprep (BioMerieux, Marcy-l'Étoile, France). All of the samples were analyzed by confocal microscopy (Zeiss LSM 880, Thornwood, NY, USA), and images were collected using ZEN-LSM software (version 2.3, Zeiss, Thornwood, NY, USA). N = 25–34 ROI from 3 animals per genotype.

### 2.4. Immunohistochemistry on Mouse Retina Cryosections

For immunohistochemistry, eyes from adult mice were enucleated, fixed in 4% PFA, and embedded in OCT. Cryosections (12 µm section) were collected and kept frozen at −80 °C until use. Cryosections were rehydrated with 1× PBS (3 × 5 min) and blocked in blocking solution (1× PBS containing 10% normal goat serum and 0.3% Triton X-100 (Sigma-Aldrich, St. Louis, MO, USA) for 1 h at room temperature. Incubation with the primary antibodies COXIV (Thermo Fisher Scientific, Rockford, IL, USA; 459600; 1:500), CERKL2, and CERKL5 (both produced in-house [5]) was performed overnight at 4 °C. After three rinses with 1X PBS (10 min each), cryosections were incubated for 1 h at room temperature with the corresponding secondary antibodies (AlexaFluor 568 anti-Mouse (Thermo Fisher Scientific, Rockford, IL, USA; A11004; 1:300) and AlexaFluor 488 anti-Rabbit (Thermo Fisher Scientific, Rockford, IL, USA; A11070; 1:300)) and with 4′,6-diamidino-2-phenylindole (DAPI) (Sigma-Aldrich, St. Louis, MO, USA; 10236276001; 1:1000). Finally, the slides were washed with 1× PBS (3 × 10 min) and coverslipped with Fluoprep (BioMerieux, Durham, NC, USA). Image visualization was performed using confocal laser scanning microscope (Zeiss LSM 880, Thornwood, NY, USA).

### 2.5. ARPE-19 Cell Culture, Transfection, and siRNA Reverse Transfection

Human ARPE-19 cells (ATCC, Elizabeth City, NC, USA; CRL_2302) were cultured in 10% fetal bovine serum (FBS) and 1% penicillin/streptomycin in 1:1 Dulbecco’s Modified Eagle’s Medium (DMEM) (ATCC, Manassas, VA, USA) and Ham’s F-12 Nutrient Mix (F12) (Life Technologies, Carlsbad, CA, USA) in a 5% CO_2_ cell culture humidified incubator at 37 °C. ARPE-19 cells were incubated without FBS for 48 h to induce differentiation.

Cell transfection was performed using Lipotransfectine (Niborlab, Guillena, Spain) (DNA–lipotransfectine ratio 1:2). ARPE-19 cells were seeded in poly-L-lysine pre-treated coverslips in 24-well plates (10^5^ cells per well) and incubated for 24 h. pEGFP-N2, CERKLb-GFP, and CERKLc-GFP vectors (1 μg per well) were transfected in non-antibiotic medium. After 5 h, the non-antibiotic medium was replaced with differentiation medium for 48 h.

For siRNA reverse transfection, cells in suspension were transfected using lipofectamine RNAiMAX reagent (Thermo Fisher Scientific, Rockford, IL, USA) and 20 nM scrambled (scr) (scr1: Non-targeting siRNA #1, D-001810-01-05, Dharmacon; and scr2: Silencer™ Negative Control No. 4 siRNA, AM4641, Ambion, Thermo Fisher, Rockford, IL, USA) or anti-CERKL small interfering (si)RNA. Anti-CERKL siRNA were obtained from Ambion (Thermo Fisher, Rockford, IL, USA), and their sequences were si*CERKL*1: 5′-UAAAACACCUGAAAAGAUAtt-3′ and si*CERKL*2: 5′-GCAUCAGAGGUCCAUAUUAtt-3. Then, ARPE-19 cells were seeded in poly-L-lysine pre-treated coverslips in 24-well plates (2 × 10^5^ cells per well) and incubated for 48 h in differentiation conditions.

Finally, in the different assays, to test the effects of oxidative stress in ARPE-19 cells, cells were treated with 100 μM sodium arsenite (NaAsO_2_) for 4 h.

### 2.6. Immunocytochemistry

After fixation with 4% PFA for 10 min at room temperature, cells were blocked with 1X PBS containing 0.01% Triton X-100 (Scharlau, Hamburg, Germany) and 10% normal goat serum or 2% sheep serum for 1 h at room temperature. Then, cells were incubated with primary antibody (anti-GFP (Abcam, Plc, Cambridge, UK; ab290; 1:1000) and anti-CERKL (in-house antibody; 1:100)) in blocking solution overnight at 4 °C and secondary antibody (AlexaFluor 488 anti-Mouse (Thermo Fisher Scientific, Rockford, IL, USA; A11017; 1:500), AlexaFluor 488 anti-Rabbit (Thermo Fisher Scientific, Rockford, IL, USA; A11070; 1:500), and Alexa Fluor 647 Phalloidin (Thermo Fisher Scientific, Rockford, IL, USA; A22287; 1:250)) with DAPI (Sigma-Aldrich, St. Louis, MO, USA; 10236276001; 1:1000) for 1 h at room temperature. Finally, coverslips were mounted using Mowiol 4-88 (Merck, Kenilworth, NJ, USA) and visualized by means of confocal microscopy (Zeiss LSM 880, Thornwood, NY, USA). Confocal images were analyzed using ImageJ software. To stain mitochondria, 1 μM MitoTracker™ Orange CMTMRos (Thermo Fisher Scientific, Rockford, IL, USA) was added to the cells and incubated for 20 min at 37 °C before fixation.

### 2.7. Mitochondrial Superoxide Quantification

MitoSOX^TM^ Red (Thermo Fisher Scientific, Rockford, IL, USA) was used to measure the mitochondrial superoxide levels. ARPE-19 cells were previously treated with 100 μM NaAsO_2_ for 4 h. Then, cells were incubated with differentiation medium containing 5 μM of MitoSOX Red at 37 °C for 10 min and washed three times with medium. Finally, cells were fixed with 4% PFA for 10 min at room temperature. Fluorescence intensity was determined by confocal microscopy (Zeiss LSM 880, Thornwood, NY, USA) and quantified by means of ImageJ software (version 1.53n, National Institutes of Health, Bethesda, MD, USA) applying CTCF (corrected total cellular fluorescence) normalization formula where cell fluorescence is corrected for the fluorescence of the background multiplied for the cell area.

### 2.8. Quantitative Analyses of Mitochondrial Morphology

Quantitative analyses of mitochondrial morphology and area in Transmission Electron Microscopy (TEM) microphotography 26,500× images and ARPE-19 cells were performed using an ImageJ software macro as described in [10]. Briefly, using ImageJ software, a threshold was established to distinguish mitochondria from the background. Particle analyses from each individual mitochondrion (particle) were performed to determine aspect ratio (AR, the ratio of the width to the height of the ellipse equal to the mitochondrion) and form factor values (FF, (4π × Am/Pm^2^), in which Am is the area of the mitochondrion and Pm is the length of the mitochondrial perimeter). The mitochondrial area in ARPE-19 cells was calculated by adding each mitochondrion area for each cell and normalized using the total area of the cell.

For TEM microphotographies, 78–89 mitochondria were analyzed from 3 WT and 3 *KD*/*KO* (3-month-old mice) and 38–44 mitochondria were analyzed from 3 WT and 3 *KD*/*KO* (12-month-old mice). Concerning ARPE-19 cells, 223–309 mitochondria were analyzed from 15–20 siRNA-treated cells per condition and 404–726 mitochondria were analyzed from 28–30 transfected cells per condition. Three experimental replicates were performed.

### 2.9. Seahorse Analysis

ARPE-19 cells were reversely transfected with scr1 and si*CERKL*1 in a 24-well plate. After 24 h, *CERKL*-silenced cells were detached and reseeded in a Seahorse 24-well cell culture plate (Agilent, Santa Clara, CA, USA) (2.5 × 10^5^ cells per well). For antioxidant and stress treatments, cells were treated with 4 μM N-acetyl-L-cysteine (NAC) (Merck, Kenilworth, NJ, USA; A9165) for 24 h and 100 μM sodium arsenite for 4 h prior to Seahorse assay. After treatments, cells were incubated with Seahorse XF Assay Medium (Agilent, Santa Clara, CA, USA) for 1 h at 37 °C. Then, plates were loaded into an XFe24 respirometry machine (Agilent, Santa Clara, CA, USA). Complex V was inhibited with 5 μM oligomycin A. Maximum oxygen consumption rate (OCR) was assayed by adding 2 μM carbonyl cyanide-p-trifluoromethoxyphenylhydrazone (FCCP). Rotenone (5 μM) and antimycin A (15 μM) were used to inhibit complex I- and III-dependent respiration, respectively. Five replicates were performed for each experimental condition.

### 2.10. Statistical Analyses

Data were analyzed using GraphPad Prism software (GraphPad6 Software Inc., San Diego, CA, USA). Homoscedasticity and normality were verified using Bartlett’s test, and D’Agostino and Person’s test, respectively. In case homoscedasticity or normality were not fulfilled, logarithm or square root corrections were performed. When data were homoscedastic and followed a normal distribution, data were analyzed by *t*-test, one-way ANOVA, and two-way ANOVA. Mann–Whitney and Kruskal–Wallis tests were used when data did not follow a normal distribution.

## 3. Results

### 3.1. KD/KO RPE Displays More Polynucleated Cells and Smaller Mitochondria

**Figure 1:**
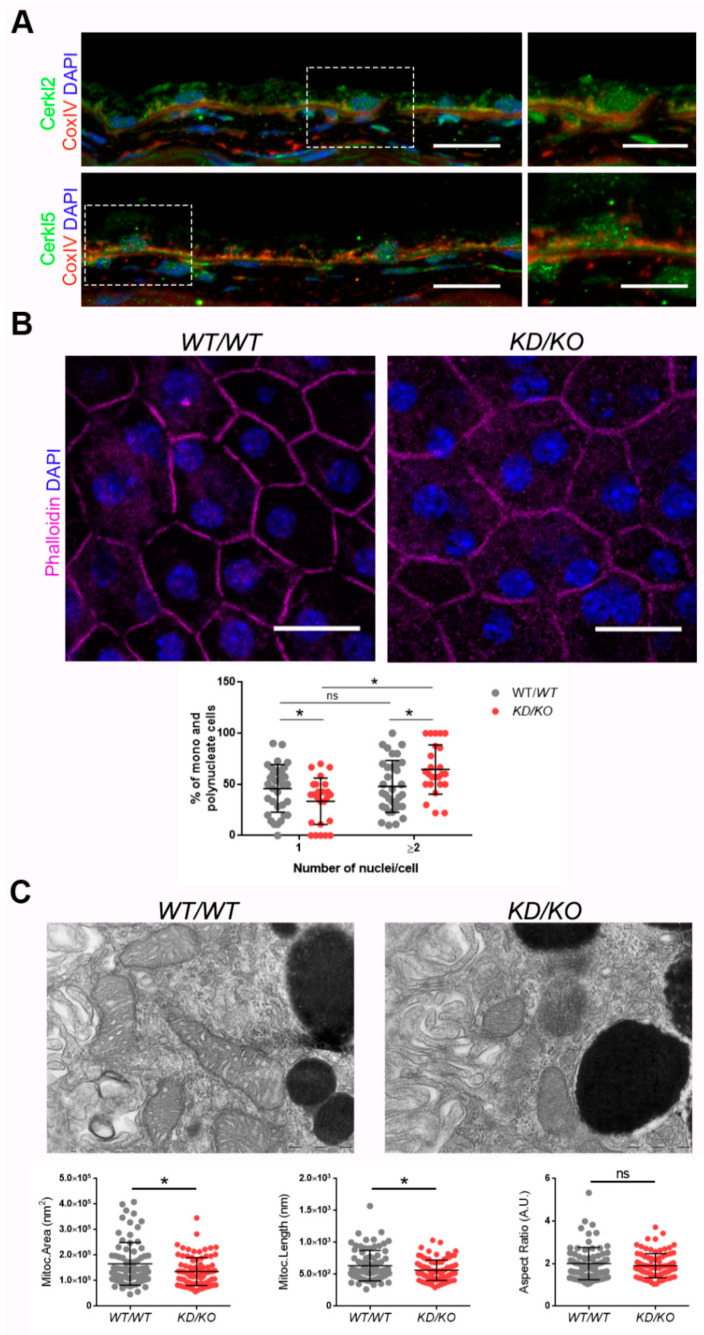
The Retinal Pigment Epithelium (RPE) of *KD*/*KO* mice shows morphological alterations. (**A**) CERKL is expressed in RPE as detected by immunofluorescence of retinal cryosections from *WT*/*WT* mice with COX-IV (red) and anti-CERKL2 or anti-CERKL5 (green) antibodies. Regions of interest (ROIs) represent higher magnification from left panels. Scale bar: 20 μm (lower magnification) or 10 μm (higher magnification). (**B**) The RPE from *KD*/*KO* mice shows an increased number of polynucleated cells. RPE flat mounts from *WT*/*WT* and *KD*/*KO* mice stained with phalloidin (for F-actin, magenta) and 4′,6-diamidino-2-phenylindole (DAPI, blue) and imaged by confocal microscopy were used to quantify the percentage of mono and polynucleated cells. Scale bar: 20 μm. (**C**) Mitochondrial fragmentation in *KD*/*KO* RPE. Transmission electron microscopy (TEM) microphotographies of retinal pigment cells from *WT*/*WT* and *KD*/*KO* (2-month-old mice) were used to quantify mitochondrial area, length, and aspect ratio. Scale bar: 500 nm. The data are expressed as the mean ± SD, *n* = 25–34 ROIs from 3 animals per group in (**B**) and *n* = 78–89 mitochondria from 3 animals per group in (**C**). Statistical analysis by Mann–Whitney test: * *p*-value ≤ 0.05.

CERKL localizes at different cellular compartments, e.g., nuclei, Golgi apparatus, and mitochondria, among others, in both immortalized and primary cell lines [8,9,10,11]. However, little is known about the subcellular localization of CERKL in mammalian RPE. To assess whether endogenous CERKL colocalizes with mitochondria in murine RPE, we performed immunofluorescence of retinal cryosections from *WT*/*WT* mice with the mitochondrial marker COX-IV and two different in-house antibodies against peptides encoded by exon 2 (anti-CERKL2) or exon 5 (anti-CERKL5), which detect different pools of endogenous isoforms [5]. In both cases, CERKL partially colocalizes with mitochondria (Figure 1A).

Detrimental factors, such as aging or oxidative stress, may compromise RPE homeostasis. Aging and blinding diseases, including rare retinopathies, are associated with changes to RPE structure, such as the increasing of bi- and multinucleated RPE cells [19,20]. Multinuclear cell formation has been proposed as a mechanism to compensate the apoptotic loss of RPE cells and to maintain the epithelial layered structure under stress conditions [21]. As *CERKL* is proposed as a resilience gene against oxidative stress in mammalian retina, and its depletion in *KD*/*KO* mice causes retinal degeneration, we investigated if *KD*/*KO* RPE suffered changes in the percentage of mono- and multinucleated cells. We observed that the ratio of mono- and poly-nucleated cells is shifted in *KD*/*KO* RPE, with an increase in poly-nucleated cells with a decrease in mono-nucleated cells (Figure 1B).

The preservation of mitochondrial function and morphology is essential to ensure RPE homeostasis and a correct crosstalk with neuroretina. Since a pool of CERKL colocalizes at mitochondria in both RPE and neuroretina, we assessed possible alterations of mitochondrial morphology in *KD*/*KO* RPE in vivo by transmission electron microscopy (TEM) of *WT*/*WT* and *KD*/*KO* RPE from 2-month-old mice. We found a significant decrease in mitochondrial area and length in *KD*/*KO*, without changes in the morphological parameter aspect ratio (Figure 1C). These findings were further confirmed by performing the same morphological analysis of mitochondria in 8-month-old mice (Figure S1A–C). On the other hand, no detectable changes in the size of the Bruch’s membrane, of which the structure is typically altered in several retinopathies, could be observed (Figure S1A,C).

Overall, our data indicated that the depletion of *Cerkl* expression alters cell structure and mitochondrial morphology in RPE cells in vivo.

### 3.2. CERKL-Silenced ARPE-19 Cells Show Alterations in Mitochondrial Network

**Figure 2:**
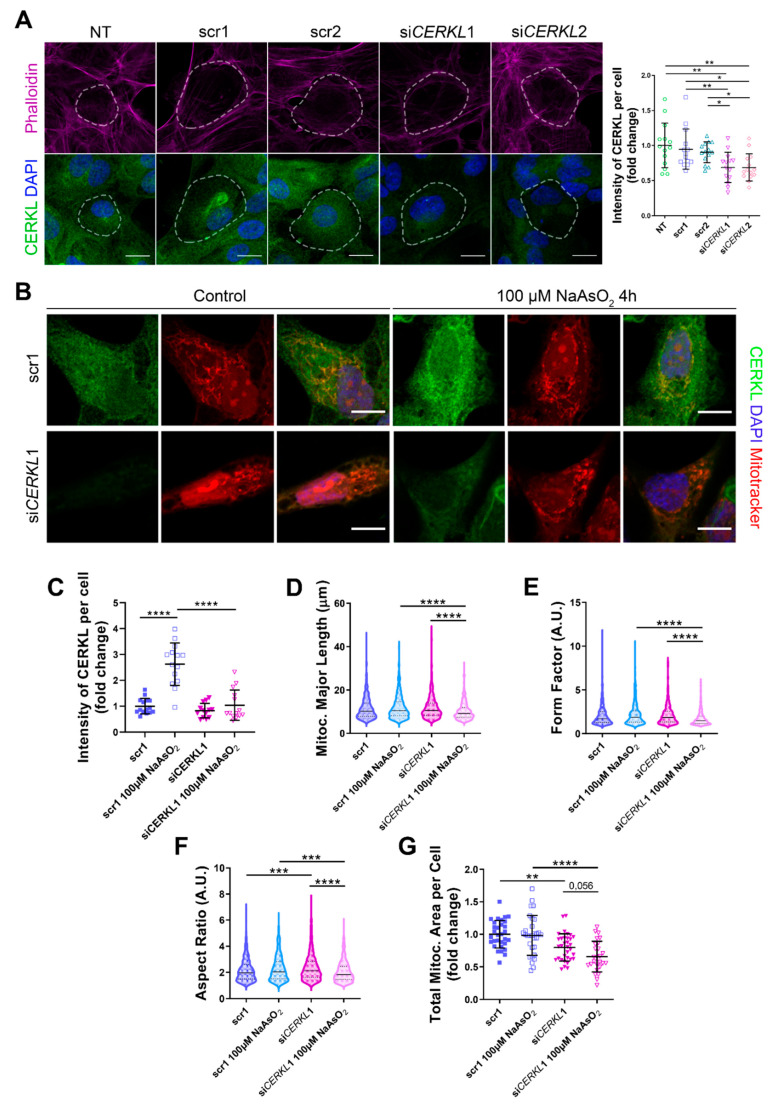
Mitochondrial morphology is altered in *CERKL*-silenced human ARPE-19 cells. (**A**) siRNAs against *CERKL* decrease *CERKL* expression in ARPE-19 cells. Immunofluorescence confocal images of non-transfected, negative control (scr1 and scr2) and *CERKL*-silenced (si*CERKL*1 and si*CERKL*2) differentiated ARPE-19 cells stained with phalloidin (magenta), CERKL (green), and DAPI (blue) were used to quantify CERKL fluorescence intensity per cell. Scale bar: 15 μm. (**B**) Mitochondrial fragmentation in *CERKL*-silenced cells under stress conditions. Immunofluorescence images from a single focal plane of negative control (scr1) and *CERKL*-silenced (si*CERKL*1) differentiated ARPE-19 cells in the control and under oxidative stress conditions were used to quantify CERKL fluorescence intensity (**C**), mitochondrial major length (**D**), form factor (**E**), aspect ratio (**F**), and mitochondrial area (**G**). Cells were stained with CERKL (green) and DAPI (blue), and mitochondria were marked with Mitotracker (red). Scale bar: 10 μm. Data are represented as the mean ± SD (**A**,**C**,**G**) and as violin plots (**D**–**F**), *n* = 15 cells per condition (**A**,**C**) and *n* = 565–997 mitochondria (**D**–**F**) from *n* = 26–33 cells (**G**) per condition. Statistical analysis by one-way ANOVA (**A**,**C**,**G**) and Kruskal–Wallis (**D**–**F**) tests: * *p*-value ≤ 0.05, ** *p*-value ≤ 0.01, *** *p*-value ≤ 0.001, **** *p*-value ≤ 0.0001.

Since depletion of *Cerkl* in *KD*/*KO* mouse model cause alterations in the mitochondrial network of RPE, we studied the deficiency of *CERKL* in vitro, taking advantage of ARPE-19 cells treated with small interference (si) RNAs against *CERKL*. To evaluate the effect of siRNAs on CERKL expression, we tested two different control siRNAs (scr1 and scr2) and two siRNAs against different regions of *CERKL* (si*CERKL*1 and si*CERKL*2) on ARPE-19 cells through immunofluorescence using an in-house antibody that recognizes most isoforms of the human CERKL protein (anti-CERKL). Phalloidin was used to delimit cell area (Figure 2A).

Quantification of CERKL fluorescence intensity revealed a significant decrease in CERKL expression in both si*CERKL*1 and si*CERKL*2-treated cells. The most significant difference in CERKL expression occurred between scr1 and si*CERKL*1 siRNAs and were thus selected for subsequent experiments (Figure 2A).

Due to genetic and environmental factors, the retina, including RPE, is constantly under stress conditions that may damage mitochondrial network organization if resilience mechanisms do not work properly [15]. To assess CERKL function in regulating the morphology of the mitochondrial network in retinal epithelium cells, we knocked down *CERKL* expression in human ARPE-19 cells using si*CERKL*1 under basal and oxidative stress conditions (100 μM sodium arsenite for 4 h) and studied mitochondrial morphology through the fluorescent mitochondrial tracker Mitotracker (Figure 2B). In accordance with previous studies, a pool of CERKL localizes at the mitochondria. Remarkably, CERKL total expression is significantly induced in ARPE-19 cells under oxidative stress conditions. Notably, this sodium arsenite-dependent boost expression of CERKL is completely impaired when *CERKL* is downregulated by si*CERKL*1 (Figure 2C).

Analyses of mitochondrial morphology revealed a significant decrease in mitochondrial major length, form factor, and aspect ratio in *CERKL*-silenced cells compared with control cells under stress conditions. In addition, these morphological parameters were significantly diminished in *CERKL*-silenced cells when comparing oxidative stress conditions with basal conditions (Figure 2D–F). Moreover, we found a significant increase in aspect ratio between control and *CERKL*-silenced cells both in basal conditions (Figure 2F). *CERKL*-silenced cells showed significantly decreased mitochondrial area in basal conditions compared with controls, and this decrease was more evident under stress conditions (Figure 2G).

To sum up, *CERKL* deficiency alters the mitochondrial network in ARPE-19 cells, particularly in response to oxidative stress conditions.

### 3.3. CERKLb and CERKLc Overexpression Restores Mitochondrial Network under Oxidative Stress in ARPE-19 Cells

*CERKL* overexpression has been described as protective against oxidative stress by interacting with antioxidant enzymes [8], regulating autophagy [9], and downregulating apoptosis [6], among others. In the human retina, due to different alternative splicing events, *CERKL* produces at least four different protein isoforms (CERKLa, CERKLb, CERKLc, and CERKLd) [6] that display different protein domains.

**Figure 3:**
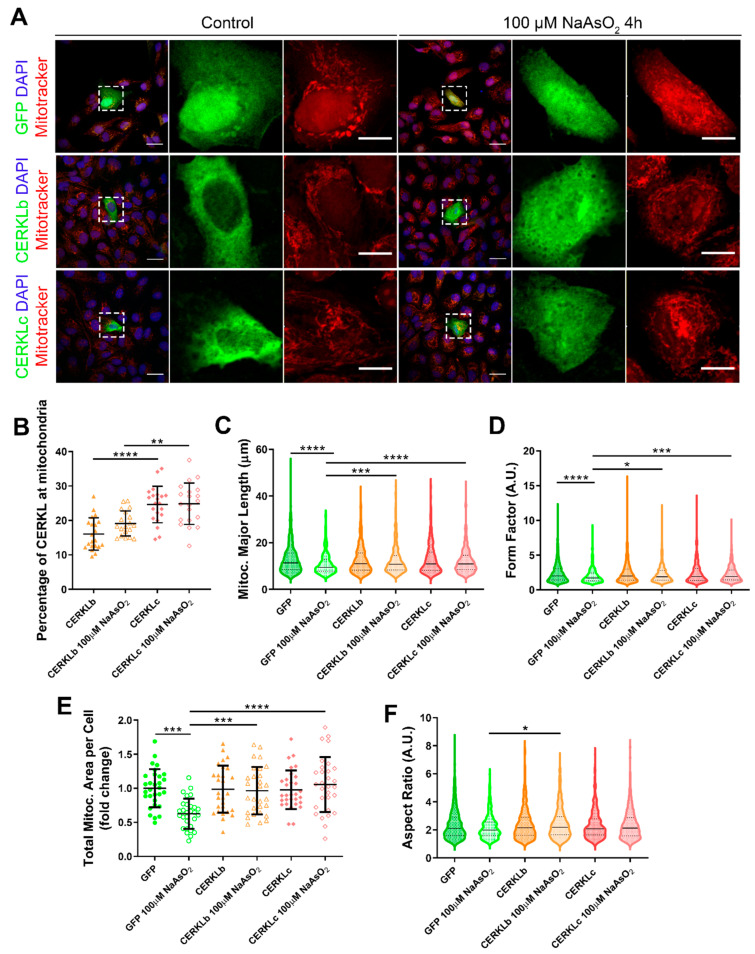
Mitochondrial morphology is restored in ARPE-19 cells under oxidative stress conditions when CERKL isoforms are overexpressed. (**A**) CERKL overexpression protects the mitochondrial network under oxidative stress conditions. Immunofluorescence images from a single focal plane of differentiated ARPE-19 cells transfected with pEGFP-N2, CERKLb-GFP, and CERKLc-GFP in the control and under oxidative stress conditions were used to quantify the percentage of CERKL at mitochondria (**B**), mitochondrial major length (**C**), form factor (**D**), mitochondrial area (**E**), and aspect ratio (**F**). Mitochondria were marked with Mitotracker (red), and nuclei were counterstained with DAPI (blue). Scale bars: 25 μm and 10 μm in higher magnification images. Data are represented as the mean ± SD (**B**,**F**) and as violin plots (**C**–**E**), n = 20–21 cells (**B**) and n = 404–726 mitochondria (**C**–**E**) from n = 28–30 cells (**F**) per condition. Statistical analysis by one-way ANOVA (**B**,**F**) and Kruskal–Wallis (**C**–**E**) tests: * *p*-value ≤ 0.05, ** *p*-value ≤ 0.01, *** *p*-value ≤ 0.001, and **** *p*-value ≤ 0.0001.

In order to test if different isoforms may differentially regulate mitochondrial dynamics, we overexpressed human CERKLb and CERKLc isoforms in ARPE-19 cells. The CERKLb isoform is the longest one (558 aa) and includes a human-specific additional exon (4b), whereas CERKLc is the shortest isoform (419 aa) and lacks the ATP-binding site and the diacylglycerol kinase (DAGK) domain. The two isoforms display a pleckstrin-homology domain (including a mRNA binding domain) and the nuclear localization and export signals. ARPE-19 cells transfected with either CERKLb or CERKLc isoforms were treated with 100 μM sodium arsenite for 4 h, and the morphology of their mitochondrial network was assessed by immunofluorescence using Mitotracker (Figure 3A). CERKLb localizes more diffusely within the cell, including the nucleus, whereas CERKLc is 10% more concentrated in mitochondria under basal and oxidative stress conditions (Figure 3A,B).

Morphological analyses showed a statistically significant decrease in mitochondrial major length, area, and form factor in GFP-transfected cells (controls) under oxidative stress compared with in the basal condition. However, mitochondria in cells transfected with both CERKLb and CERKLc did not show any significant change in these parameters between basal and oxidative stress conditions. In fact, the mitochondrial length, area, and form factor in CERKLb and CERKLc-transfected cells under stress conditions were not affected in contrast with the decrease in all these measurements in stressed control cells (Figure 3C–F).

Altogether, these results strongly indicated that both CERKLb and CERKLc localized at mitochondria and that CERKL overexpression in ARPE-19 cells protected mitochondrial network under oxidative stress conditions.

### 3.4. Mitochondrial Superoxide Production Is Reduced by CERKLb and CERKLc Overexpression in ARPE-19 Cells

**Figure 4:**
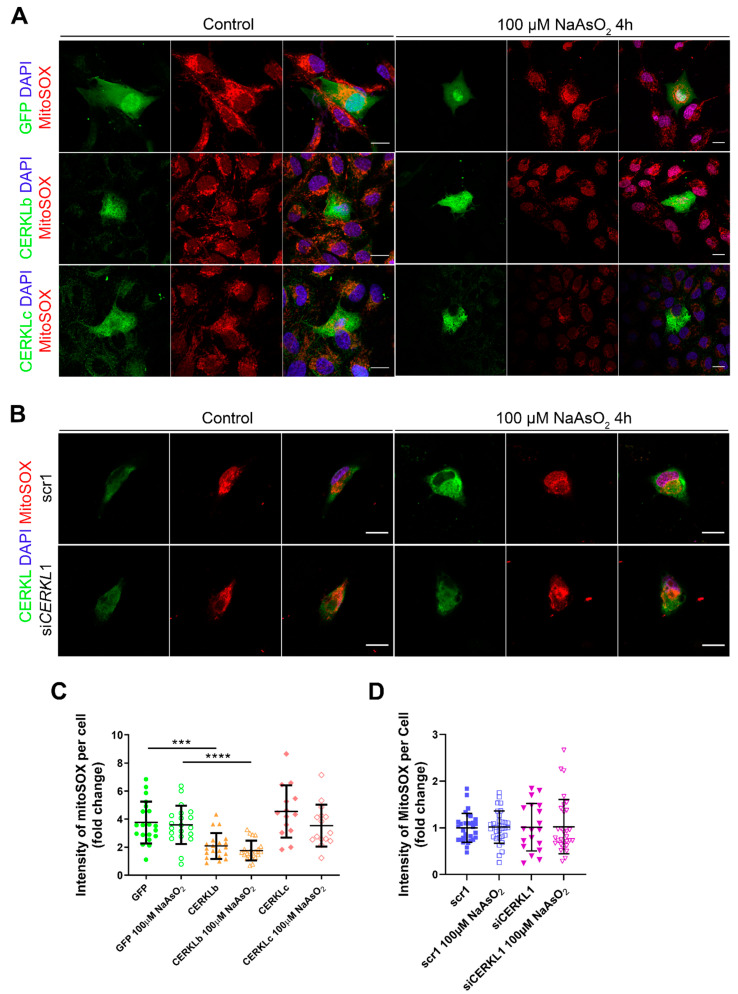
Quantification of MitoSOX in ARPE-19 cells overexpressing CERKLb and CERKLc or knocking down *CERKL*. (**A**) Overexpression of CERKLb and CERKLc reduces the production of mitochondrial superoxide. Immunofluorescence images from a single focal plane of differentiated ARPE-19 cells transfected with pEGFP-N2, CERKLb-GFP, and CERKLc-GFP under control and stress conditions were used to quantify MitoSOX fluorescence intensity (**C**). Mitochondrial superoxide was detected with MitoSOX (red), and nuclei were counterstained with DAPI (blue). Scale bar: 15 μm. (**B**) *CERKL* depletion is not sufficient to increase mitochondrial superoxide production under basal and stress conditions. Immunofluorescence images from a single focal plane of negative control (scr1) and *CERKL*-silenced (si*CERKL*1) differentiated ARPE-19 cells under basal and oxidative stress conditions were used to quantify MitoSOX fluorescence (**D**). Cells were stained with CERKL (green) and DAPI (blue), and mitochondrial superoxide was detected with MitoSOX (red). Scale bar: 15 μm. Data are represented as the mean ± SD, *n* = 28–33 cells per condition. Statistical analysis by Kruskal–Wallis test: *** *p*-value ≤ 0.001 and **** *p*-value ≤ 0.0001.

Mitochondrial network disorganization is a typical sign of cellular damage and loss of mitochondrial homeostasis [22]. Under oxidative stress or pathogenic conditions, damaged mitochondria generate free radicals, such as mitochondrial superoxide, due to impaired antioxidant response [23]. Therefore, we measured mitochondrial oxidation using MitoSOX quantification in cells either overexpressing CERKLb and CERKLc or depleted in *CERKL* expression under both control and oxidative stress conditions (Figure 4A,B). A significant decrease in MitoSOX intensity was observed when overexpressing CERKLb and CERKLc in basal conditions, and this mitochondrial protection was maintained under oxidative stress (Figure 4C). On the other hand, neither control cells nor cells depleted in *CERKL* showed an increase in MitoSOX in any condition, thus pointing to other factors being necessary for mitochondrial superoxide production besides stress by sodium arsenite (Figure 4D).

In summary, these findings pointed to a protection of mitochondria as measured by mitochondrial superoxide production when CERKLb and CERKLc were overexpressed.

### 3.5. CERKL Knock-Down Alters Mitochondrial Respiration

Considering that (i) *CERKL* deficiency affects mitochondrial network organization in ARPE-19 cells, and (ii) oxygen consumption and other mitochondrial energy-related measurements in neural retinas of *KD*/*KO* mice are compromised, we performed Seahorse analysis to evaluate mitochondrial function and oxygen consumption rate (OCR) in *CERKL*-depleted ARPE-19 cells. We compared mitochondrial performance in control and stress conditions as well as under antioxidant pre-treatment (4 μM N-acetyl-L-cysteine (NAC) for 24 h).

**Figure 5:**
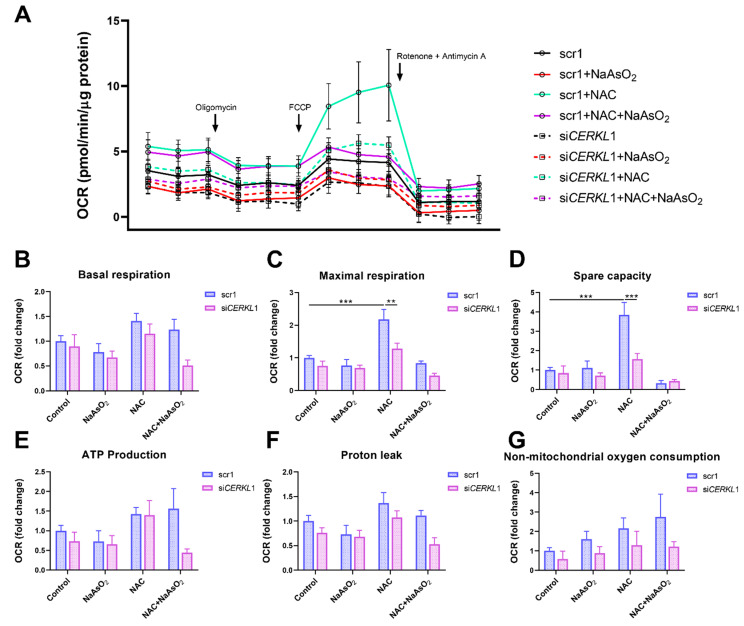
Oxygen consumption is impaired in *CERKL*-depleted ARPE-19 cells under different antioxidant (NAC pre-treatment) and oxidative stress (NaAsO_2_ treatment) conditions. (**A**) Altered oxygen consumption rate (OCR) in *CERKL*-knocked-down cells in all conditions; straight lines indicate values from scrambled control cells, whereas dotted lines indicate si*CERKL* treated cells. (**B**) Basal respiration are the initial OCR levels without treatment. (**C**) Maximal respiration is obtained after the addition of FCCP (carbonyl cyanide-4-trifluoromethoxy phenyl-hydrazone). (**D**) Spare capacity is calculated by subtracting basal respiration from maximal respiration. Note that *CERKL*-depleted cells neither increase the respiratory capacity after antioxidant treatment nor maintain maximal respiration and spare capacity after oxidative injury. Oligomycin addition reveals (**E**) ATP production and (**F**) proton leak levels. (**G**) Non-mitochondrial oxygen consumption values are obtained after rotenone and antimycin A addition. Statistical test by two-way ANOVA: ** *p*-value ≤ 0.01, *** *p*-value ≤ 0.001.

Differentiated ARPE-19 cells displayed low oxygen consumption rates, which made changes between conditions very subtle. Under control conditions, OCR was reduced (black straight versus dotted lines) upon knock-down of *CERKL* expression (Figure 5A). In fact, all respiration-related parameters, including maximal respiration and non-mitochondrial oxygen consumption, showed a clear trend of diminishing in *CERKL*-deficient cells (Figure 5B–G). As expected, we found a decrease in the respiration parameters (basal and maximal respiration) after NaAsO_2_ treatment in both scr1 and si*CERKL*1-transfected cells, conserving the tendency.

NAC pre-treatment results in an increase (two-fold) in mitochondrial and non-mitochondrial respiration in control cells, which allows them to resist oxidative stress conditions. However, *CERKL*-depleted cells are not able to respond to the antioxidant pre-treatment to these high levels and are thus not resilient to oxidative injury. Remarkably, mitochondrial respiration is decreased in *CERKL*-depleted cells, while non-mitochondrial oxygen consumption increases slightly in all conditions, probably as a cell compensatory mechanism.

Overall, these results denoted that *CERKL*-deficient ARPE-19 cells do not respond to antioxidant (NAC) protective pre-treatment and are thus not as resilient to oxidative stress as control cells.

## 4. Discussion

Mutations in many different genes cause retinal degeneration. Most of them affect specific photoreceptor functions (e.g., phototransduction), but mutations in genes expressed ubiquitously (e.g., splicing genes) or in the RPE (such as *RPE65*) can also alter photoreceptor homeostasis and lead to retinal cell death (reviewed in [24]). The correct function of RPE is crucial for photoreceptor survival, but how mutations in photoreceptor genes alter RPE is not well documented. However, dysfunctional proteins can have both an impact in photoreceptors and RPE, thus contributing to the retinal degeneration phenotype. In fact, patients carrying mutations in *CERKL* present early onset rod-cone dystrophy despite relatively preserved visual acuity and a very distinctive RPE phenotype, e.g., RPE granularity and frank macular RPE atrophy [25]. A *cerkl* knock-out zebrafish model also shows defects in photoreceptor phagocytosis due to the dysfunction of RPE [26]. Although the precise function of CERKL is not yet fully determined, several reports associate CERKL with basic cell functions related to stress resilience, such as autophagy [9], stress granule production [11], and mitochondrial physiology and dynamics [10]. Within this context, we aimed to explore the alteration of RPE due to *CERKL* mutations both in vivo in a *Cerkl* mouse model and in vitro, using cultured cells to study the effect of *CERKL* overexpression and knock-down on the mitochondrial network organization.

This work showed that mitochondria in the RPE of the *Cerkl^KD/KO^* mouse (which expresses very low *CERKL* levels) show a significant decrease in length and area, in full agreement with previous results observed in photoreceptors and retinal ganglion cells [10], which reported an increase in mitochondrial fragmentation. To further dissect the contribution of CERKL to mitochondrial dynamics, particularly under oxidative stress conditions, we performed in vitro assays. The addition of sodium arsenite and other reagents to culture media is commonly used as a proxy for physiological oxidative stress conditions, as the cell effects can be easily quantified and compared with basal conditions [27], even though it might not elicit the full retinal cell response to injury.

Our results showed that the knock-down of endogenous *CERKL* levels in ARPE-19 cells reproduced the same results as observed in vivo, with cells displaying mitochondria with an altered shape and decreased length. This fragmentation effect was more apparent in cells under oxidative stress conditions, which presented around 20% less of mitochondrial area per cell in *CERKL*-depleted cells (Figure 2G). Although the mitochondrial network is altered, the production of superoxide radicals in mitochondria is not affected in any condition, thus indicating that at least in ARPE-19 cells, sodium arsenite does not induce superoxide production as an early response, irrespective of CERKL levels. Other reports claim that sodium arsenite is required but is not sufficient to induce the full cellular response to oxidative stress [28]. Notably, control cells under oxidative stress showed a 2.5-fold increase in endogenous CERKL protein expression but knocked-down cells were unable to induce this transcriptional response to injury. In this context, depletion of *CERKL* decreases the oxygen consumption rate, but this decrease is more prominent under oxidative stress conditions: in fact, *CERKL*-depleted cells are not responsive to antioxidants protective treatment, indicating that *CERKL* expression is associated with oxidative stress resilience and that this lack of *CERKL* hampers antioxidant-dependent response. These results are in agreement with the mitochondrial oxygen consumption impairment in the neural retinas of *Cerkl^KD/KO^* mice.

As mentioned, *CERKL* produces a high number of alternatively spliced transcripts that encode differential protein domains. To explore the potential protection against oxidative stress of the mitochondrial network due to *CERKL* expression, we analyzed the effects of overexpressing either the longest (558 aa) or the shortest protein isoform (419 aa) in ARPE-19 cells. When challenged by oxidative stress and in contrast with what happens in controls and in cells depleted in *CERKL*, cells overexpressing either isoform show healthy mitochondria in which the length and aspect are maintained, at least after 4 h of treatment. In fact, under stress, the total mitochondrial area per cell remains unaffected in cells transfected with CERKL constructs, whereas control cells show a reduction to half of the mitochondrial mass (Figure 3E).

**Figure 6:**
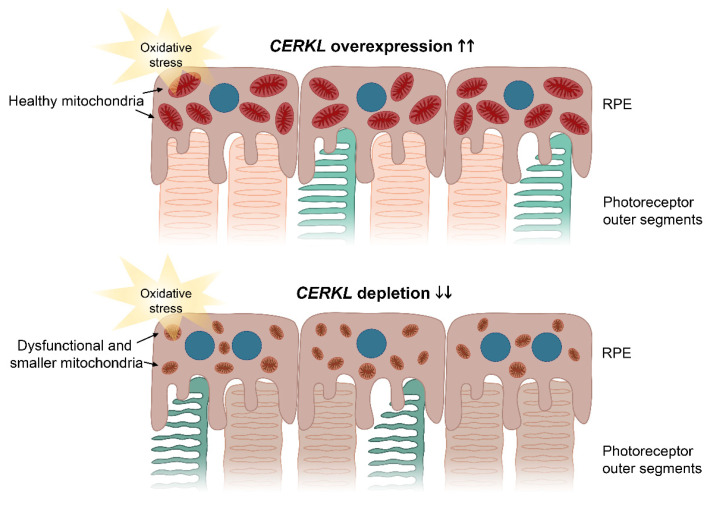
Image depicting the effects of *CERKL* overexpression and depletion in mitochondrial shape and size in RPE cells upon oxidative stress conditions. Under oxidative stress, *CERKL* overexpression (**upper panel**) protects RPE mitochondrial morphology, whereas depletion in *CERKL* (**lower panel**) causes mitochondrial fragmentation, respiratory alterations, and polynucleation in RPE. Dysfunction of RPE affects photoreceptor homeostasis and can lead to retinal degeneration. Blue—nuclei; brownish red—healthy mitochondria; brown—dysfunctional and smaller mitochondria.

These protective effects of CERKL overexpression upon mitochondrial physiology in the RPE were also supported by the analysis of damaging superoxide radicals in mitochondria, which was half that of controls in any condition. These results complement previous work on retinal neurons showing that the depletion of *CERKL* causes mitochondrial fragmentation and a substantial decrease in mitochondrial metabolism [10]. The resulting model based on our results is shown in Figure 6.

Our results further reinforce the role of *CERKL* as a resilient gene, since oxidative stress treatment induces the expression of CERKL as a very early cell response: we observed a more than two-fold increase in endogenous CERKL 4 h after the addition of the arsenite reagent. On the other hand, the subcellular localization of the two CERKL isoforms is subtly different and changes depending on the perceived injury by oxidative stress. The shortest isoform (which does not display the DAGK domain) presents stronger mitochondrial localization than the longest isoform (25% versus 15% of the CERKL pool, respectively), with the latter localizing all over the cell, including the nucleus. Previous reports showed that the longer isoforms of CERKL increase the nuclear localization upon oxidative stress and formed part of stress granules [11]. We further complement these results by showing that the overexpression of CERKL protects mitochondria against oxidative injury.

## 5. Conclusions

Overall, our results suggest that one of the physiological roles of CERKL is to protect the mitochondrial network in retinal cells, both neurons and epithelium, against the injury of light/oxidative stress. In this context, some unusual and particular phenotypic traits of the RPE observed in the retinas of patients carrying *CERKL* mutations, may reflect the combination of photoreceptor and RPE alterations.

Finally, we propose that the high level of physiological interplay between photoreceptors and RPE cells warrants including RPE assessment in the clinical phenotype of RP and other retinal dystrophy patients. Mutations in many RP genes, such as *CERKL*, can potentially affect both tissues. By identifying RPE pathogenic events, particularly those related to oxidative stress, we could design specific drug- or cell-based therapies that target RPE to improve photoreceptor homeostasis and/or to halt photoreceptor neurodegeneration.

## Data Availability

The data presented in this study are available in this article and supplementary material.

## Supplementary Materials

The following are available online at https://www.mdpi.com/article/10.3390/antiox10122018/s1, Figure S1: Mitochondrial fragmentation in RPE from 18-month-old *KD*/*KO* albino mice.
