# Supplementary material for: Overexpression of CERKL Protects Retinal Pigment Epithelium Mitochondria from Oxidative Stress Effects
Source: Antioxidants (Basel). 2021 Dec 19;10(12):2018. doi: 10.3390/antiox10122018 (PMC8698444; doi:10.3390/antiox10122018)
Supplement: Supplementary file 1 [file antioxidants-10-02018-s001.zip › antioxidants-1511560-supplementary.pdf]

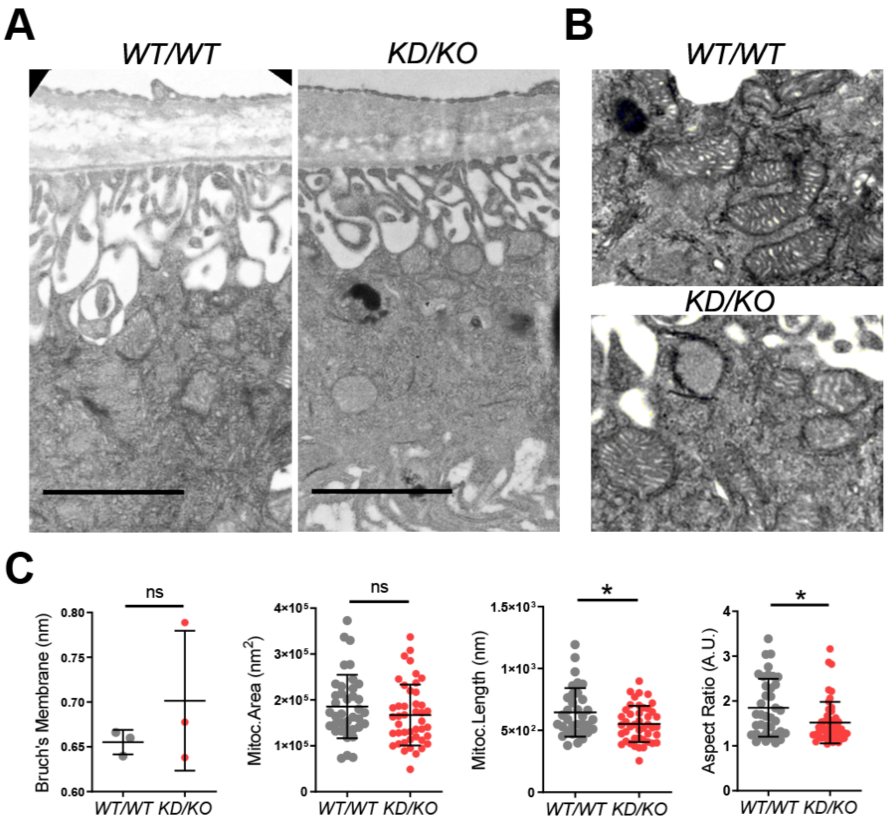

Figure S1. Mitochondrial fragmentation in RPE from 18-month-old *KD/KO* albino mice. (**A-B**) TEM microphotographies of retinal pigmented cells from *WT/WT* and *KD/KO* of old mice (18 months) were used to quantify different aspects of mitochondria. (**C**) Graphics of Bruch's membrane width, and mitochondrial area, length, and aspect ratio. Scale bar: 500 nm. The data are expressed as the mean ±SD, n=38-44 mitochondria from 3 animals per group. Statistical analysis by Mann-Whitney test: *p-value ≤ 0,05.
